# Emotion Recognition Based on Weighted Fusion Strategy of Multichannel Physiological Signals

**DOI:** 10.1155/2018/5296523

**Published:** 2018-07-05

**Authors:** Wei Wei, Qingxuan Jia, Yongli Feng, Gang Chen

**Affiliations:** School of Automation, Beijing University of Posts and Telecommunications, Beijing 100876, China

## Abstract

Emotion recognition is an important pattern recognition problem that has inspired researchers for several areas. Various data from humans for emotion recognition have been developed, including visual, audio, and physiological signals data. This paper proposes a decision-level weight fusion strategy for emotion recognition in multichannel physiological signals. Firstly, we selected four kinds of physiological signals, including Electroencephalography (EEG), Electrocardiogram (ECG), Respiration Amplitude (RA), and Galvanic Skin Response (GSR). And various analysis domains have been used in physiological emotion features extraction. Secondly, we adopt feedback strategy for weight definition, according to recognition rate of each emotion of each physiological signal based on Support Vector Machine (SVM) classifier independently. Finally, we introduce weight in decision level by linear fusing weight matrix with classification result of each SVM classifier. The experiments on the MAHNOB-HCI database show the highest accuracy. The results also provide evidence and suggest a way for further developing a more specialized emotion recognition system based on multichannel data using weight fusion strategy.

## 1. Introduction

Emotion recognition is a quickly developing branch of affective computing, which is integration of psychology, physiology, computer science, and so on. And various studies have shown that emotion plays a vital role in artificial intelligent [1]. Emotion recognition enables computer to provide the appropriate feedback to emotion state of human, which can be applied to various applications, such as learning environment, leisure entertainment, medical assist, and mental health [2–5]. For example, if computers have ability to detect emotion of student and create an appropriate feedback according to the emotion state, the student can be more effective in online learning environment.

It is imperative to take into account physiological signals to recognize emotion because of the strong relationship between physiological reactions and human. Besides, physiological signals are the result of Central Nervous System (CNS) and Autonomic Nervous System (ANS) activities, which are the same among people with different cultures, languages, and gender and cannot be imitated easily [6]. And physiological activations are largely involuntary which cannot be triggered by any conscious or intentional control easily [7].

Since the complexity of body structure, various physiological activities are related to emotional state. The corresponding various physiological signals can be used for emotion recognition, such as EEG, ECG, GSR, RA, and Blood Volume Pressure (BVP). Single channel physiological signals presented do have some limitations; therefore the emotion recognition was proposed based on multichannel physiological signals. Relevant researches have achieved a certain level of development. However, the difference between various physiological signals has not been considered adequately. It is motivated by the fact that the strength of expression for physiological signal on various emotions is different. We propose a new principal of weight design based on feedback strategy, which uses signal emotional state recognition rate based on single physiological signal to calculate the weight matrix of each physiological signal.

For our proposed method, there are three main contributions. (1) In the weight definition stage, a more advanced strategy based on feedback is used. In construction, physiological signal expounds the expressiveness of emotional state by the recognition rate of each emotional state based on each physiological signal. Then we calculate weight matrix of each classifier based on single physiological signal. (2) In the fusion stage, a more advanced weight fusion strategy is used in decision level. We linearly fuse weight matrix with classification result of each classifier. And max-win strategy is used for final emotion recognition result. (3) The proposed method has been evaluated in a database which contains multichannel physiological signals. Moreover, comparison results have been carefully analyzed and studied on whether to use weight matrix based on strategy of feedback or not. The rest of the paper is organized as follows: Section 2 gives an overview of related works on emotion recognition based on multichannel physiological signals.  Section 3 describes the materials and methods in use.  Section 4 verifies the proposed method by experiment and analyzes experimental results.  Section 5 concludes the paper.

## 2. Related Work

In the past long time, the issue of defining and describing emotion states has been a constant challenge in different subjects of the behavioral and social sciences. The discrete emotional model proposed by Ekman [8] and two-dimensional continuous emotional model proposed by Lang [9] are generally used in emotion recognition research. In order to improve the use of emotion classification algorithm, discrete emotional model is the mostly adopted model in the current study. In the discrete emotional model, several basic emotions are considered separately since they do not have common attributes. The other emotions are considered a mix of these basic emotions. Ekman [8] proposed six discrete basic emotions, which contain happiness, sadness, surprise, anger, disgust, and fear which were considered in our study.

Most researchers divide human physiological signal sources into two categories: brain activities and peripheral physiological activities. Brain activities are measured by EEG, Magnetoencephalography (MEG), functional near-infrared spectroscopy (fNIRS), functional magnetic resonance imaging (fMRI), etc. On the other hand, peripheral physiological activities are measured by ECG, heart rate, Electromyography (EMG), BVP, GSR, RA, finger temperature, etc. The commonly used EEG signals reflect emotion changes on the CNS, while the peripheral signals reflect the emotion influence on the ANS. From the clinical point of view EEG [10], ECG [11], GSR [12], and RA [13] are most widely used physiological signals for emotion recognition. Various physiological signals can be fused together to determine and classify various kinds of emotion [14]; therefore the focus of research turns to multimodal information fusion.

Previous works on fusion strategies can be broadly categorized into feature level fusion and decision-level fusion. Feature level fusion aims to directly combine feature vectors by concatenation [15] or kernel methods [16]. Decision-level fusion combines the prediction scores of each single classifier. The advantage of decision-level fusion is that it can combine different types of classifiers like logistic regression and SVM [17]. Previous works usually conducted it by a single layer averaging [18] or weighted voting [19]. For fusion strategy, we adopt weighted decision-level fusion strategy. Calculating weight is the key element that gives a weight to various features based on certain principles.

Previous various approaches on emotion have reported a correlation between basic emotions and physiological responses. Usually different classifiers such as hidden Markov models (HMMs [20]), k Nearest Neighbors (k-NN) algorithm [21], SVM [22], support vector regression (SVR) [23], and linear discriminant analysis (LDA) [24] have been used for emotion recognition based on physiological signals. For classifier we chose a SVM, as they have previously been proven to be very effective and to maintain enough flexibility with regard to their main parameter optimization [25, 26]. And SVM have been reported in literature as obtaining the highest classification results when using multidimensional data [27].

## 3. Materials and Methods

### 3.1. Emotion Feature Extraction and Selection

Physiological signals are highly dimensional data which may contain a lot of useless features. Therefore, the most important thing of emotion recognition system is extracting appropriate and efficient features of physiological signals. Various analysis domains have been used in physiological emotion features extraction, including time, frequency, and statistical analysis. Time domain analysis is based on the geometric properties of physiological signals, such as amplitude, mean value, and variance. As the earliest method applied by researchers, the advantage is its simplicity and intuition. Frequency domain analysis is based on the character of every frequency. The most widely used method is power spectrum estimation, which obtains correspondence between power and the frequency by signal conversion. Time-frequency domain analysis is based on the comprehensive analysis of characters of both time and frequency domain features. In this study, we consider the combination of the time and frequency domain features for the physiological signals.

**Figure 1:**
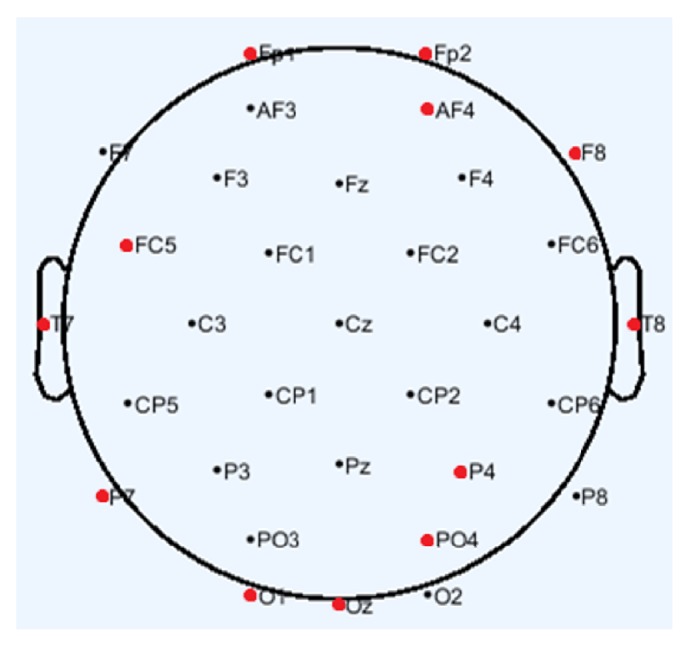
EEG electrode locations.

EEG is an electrophysiological monitoring method to record electrical activity of the brain. The MAHNOB-HCI database provides EEG data recorded from 32 channels, 14 of which were from left hemisphere, 14 from right hemisphere, and 4 from midline. Researches have indicated that the emotion perception in human brain requires coordination between different brain regions. The main lobes involved in emotion perception include frontal lobes, temporal lobes, and parietal lobes [28]. Besides, the necessity of channel selection in EEG-based emotion recognition has been testified [29]. Therefore, the selected 12 channels are Fp1, FC5, T7, P7, and O1 from left hemisphere, Fp2, AF4, F8, T8, P4, and PO4 from right hemisphere, and Oz from midline which are shown in Figure 1. In general, bands of frequency for each EEG channel correspond to delta (0-4 Hz), theta (4-7 Hz), alpha (8-15 Hz), and beta (16-31 Hz) [30]. Delta and theta are seen in babies and young children normally. Alpha emerges with closing of the eyes. Beta is seen on both sides in symmetrical distribution and most evident frontally. Therefore, the selected band of frequency is beta. Multiple types of EEG features have been used in emotion recognition, including both aspects of frequency domain and time domain features. Above all, we select mean, standard, and max of power spectral density of the beta to form 36-dimensional EEG feature vector as follows:

(1) $$\begin{array}{c} {\vec{F}}_{1}=\left(\;f_{11},f_{12},\ldots ,f_{1,36}\;\right). \end{array}$$

**Figure 2:**
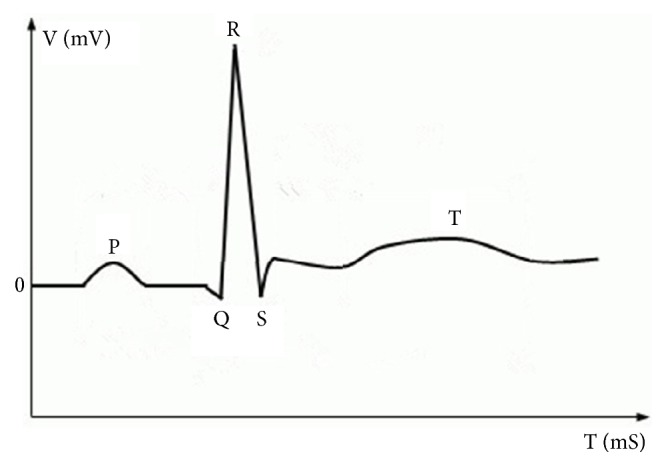
Typical structure of ECG signal.

Electrocardiography (ECG) signal is a measure of electrical activity associated with the heart. The MAHNOB-HCI database provides ECG data recorded from 3 channels. ECG signals have been defined in medicine strictly which produces four entities: P wave, QRS complex wave, T wave, and U wave and each has a unique pattern [31] which are shown in Figure 2. The P wave represents atrial depolarization. The QRS complex wave represents ventricular depolarization. The T wave represents ventricular repolarization. The U wave represents papillary muscle repolarization. Additionally, heart rate variability (HRV) is the physiological phenomenon of variation in the time interval between heartbeats. It represents fluctuation between adjacent R where R is a point corresponding to the peak of the QRS complex wave. The amplitude peak of P, R, T wave, and HRV can describe the characteristics of signal with the changes of emotion states. And multiple types of ECG features have been used in emotion recognition, including both aspects of frequency domain and time domain features. Therefore, we select mean and standard of amplitude of P, R, T, and HRV in time domain. Besides, we select max, mean, and standard of power spectral density of HRV in frequency domain. Above all, we get 33-dimensional ECG feature vector as follows:

(2) $$\begin{array}{c} {\vec{F}}_{2}=\left(\;f_{21},f_{22},\ldots ,f_{2,33}\;\right). \end{array}$$

Respiration amplitude (RA) is commonly acquired by measuring the physical change of the thoracic expansion with a rubber band around the chest or belly [13]. In general, relaxation and startling event causes rate decreases, tense situations may result in momentary cessation, and negative emotions generally cause pattern irregularity. Besides, RA is closely linked to heart function. The MAHNOB-HCI database provides RA data recorded from one channel. The first difference and second difference of RA can also describe the characteristics of signal with the changes of emotion states. And multiple types of RA features have been used in emotion recognition, including both aspects of frequency domain and time domain features. Therefore, we select median, mean, variance, minimum, maximum, and maximum and minimum difference of RA in both time and frequency domains. Besides, we select median, mean, variance, minimum, maximum, and maximum and minimum difference of first and second difference of RA in time domains. Above all, we get 28-dimensional ECG feature vector as follows:

(3) $$\begin{array}{c} {\vec{F}}_{3}=\left(\;f_{31},f_{32},\ldots ,f_{3,28}\;\right). \end{array}$$

Galvanic skin response (GSR) is physically the measure of change in the electrical properties of the skin [32] in response to changes in the ANS. Sympathetic activity causes increase in the sweat gland activity leading to a decrease in the level of skin resistance. Thus GSR is a manifestation of the sympathetic activity or emotional arousal. Specific emotions cannot be accurately identified because some emotions produce similar GSR responses, like anger and startle response [33]. However, GSR has high importance in emotion recognition clubbed with other physiological signal such as ECG. The MAHNOB-HCI database provides GSR data recorded from one channel. The first difference and second difference of GSR can also describe the characteristics of signal with the changes of emotion states. And multiple types of GSR features have been used in emotion recognition, including both aspects of frequency domain and time domain features. Therefore, we select median, mean, variance, minimum, maximum, and maximum and minimum difference of GSR in both time and frequency domains. Besides, we select median, mean, variance, minimum, maximum, and maximum and minimum difference of first and second difference of GSR in time domains. Above all, we get 28-dimensional GSR feature vector as follows:

(4) $$\begin{array}{c} {\vec{F}}_{4}=\left(\;f_{41},f_{42},\ldots ,f_{4,28}\;\right). \end{array}$$

### 3.2. SVM Classification

Following the extraction of features, a classifier is trained to recognize emotion states. Nonlinear SVM was evaluated in this study. SVM is a new supervised learning model with associated learning algorithm for classification problem of data whose ultimate aim is to find the optimal separating hyperplane. The mathematical model of SVM is shown as below.

Given a training set ${\left\{y_{i},{\vec{x}}_{i}\right\}}_{i=1}^{l}$, where ${\vec{x}}_{i}\in R^{n}$ is input and *y*_*i*_ ∈ {−1, +1} is the corresponding output, if there is a hyperplane which can divide the all the points ${\vec{x}}_{i}$ into two groups correctly, we aim to find the “maximum-margin hyperplane” where the distance between the hyperplane and the nearest point ${\vec{x}}_{i}$ from either group is maximized. By introducing the penalty parameter *c* > 0 and the slack variable $\vec{\xi }=\left(\xi_{1},\xi_{2},\ldots ,\xi_{l}\right)$, the optimal hyperplane can be obtained by solving constraint optimization problem as follows:

(5) min 12ω→2+C∑i=1lξis.t. yiω→·x→i+b≥1,ξi≥0,  i=1,2,…,l

Based on Lagrangian multiplier method, the problem is converted into a dual problem as follows:

(6) max ∑i=1lai−12∑i,j=1laiajyiyjx→i·x→js.t. ∑i=1laiyi=0,0≤ai≤C,  i=1,2,…,l

where *a*_*i*_ > 0 are the Lagrange multipliers of samples ${\vec{x}}_{i}$. Only a few *a*_*i*_ > 0 are solutions of the problem of removing the parts of *a*_*i*_ = 0, so that we can get the classification decision function as follows:

(7) $$\begin{array}{c} f\left(\;x\;\right)=\mathrm{sign}\left[\;\overset{l}{\underset{i=1}{\sum }}a_{i}y_{i}\left(\;{\vec{x}}_{i}\cdot \vec{x}\;\right)+b\;\right] \end{array}$$

For the linearly nonseparable problem, we first map the data to some other high-dimensional space *Η*, using a nonlinear mapping which we call Φ. Then we use linear model to achieve classification in new space *Η*. Through defined “kernel function” *Κ*, (6) is converted as follows:

(8) max ∑i=1lai−12∑i,j=1laiajyiyjkx→i·x→js.t. ∑i=1laiyi=0,0≤ai≤C,  i=1,2,…,l

And the corresponding classification decision function is converted as follows:

(9) $$\begin{array}{c} f\left(\;x\;\right)=\mathrm{sign}\left[\;\overset{l}{\underset{i=1}{\sum }}a_{i}y_{i}k\left(\;{\vec{x}}_{i}\cdot \vec{x}\;\right)+b\;\right] \end{array}$$

The selection of kernel function aims to take the place of inner product of basis function. The ordinary kernel functions investigated for linearly nonseparable problems are as follows:

(1) nth-degree polynomial kernel function is

(10) $$\begin{array}{c} k\left(\;{\vec{x}}_{i},{\vec{x}}_{j}\;\right)={\left(\;{\vec{x}}_{i}\cdot {\vec{x}}_{j}+1\;\right)}^{d},\;d=1,2,\ldots \end{array}$$

(2) (Gaussian) radial basis kernel function is

(11) $$\begin{array}{c} k\left(\;{\vec{x}}_{i},{\vec{x}}_{j}\;\right)=\exp\left(\;-\gamma {\left\|\;{\vec{x}}_{i}-{\vec{x}}_{j}\;\right\|}^{2}\;\right) \end{array}$$

(3) Sigmoid kernel function is

(12) $$\begin{array}{c} k\left(\;{\vec{x}}_{i},{\vec{x}}_{j}\;\right)=\tanh\left[\;b\left(\;{\vec{x}}_{i}\cdot {\vec{x}}_{j}\;\right)+c\;\right],\;b>0,\;\;c<0 \end{array}$$

In this study, we used RBF kernel function. And grid search method was applied to optimize the parameters *γ* and *c*.

### 3.3. Weighted Fusion Strategy

**Figure 3:**
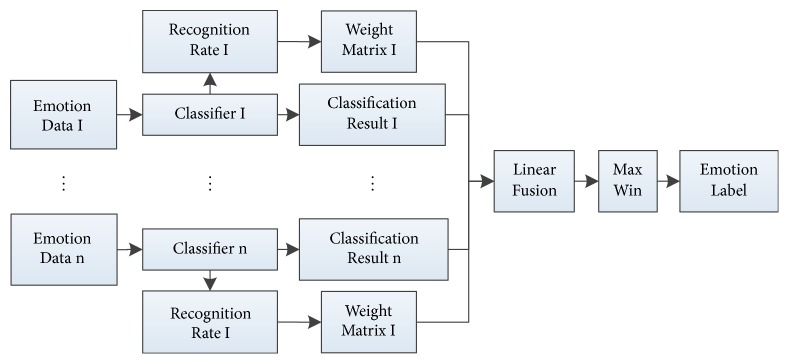
Flow of weight fusion strategy.

In this section, we propose a novel decision-level weight fusion network as shown in Figure 3 to combine the results of each independent classifier. Weighted fusion strategy is based on weight matrix, which is defined as Definition 1.

**Definition 1 .**

Let *W* be a linear transformation square matrix of order *n*, where *n* is the number of categories. The different choices for *W* lead to different weight situation.

(1)  *W* is an identity matrix of order *n*, which is no weight situation.

(13) $$\begin{array}{c} W=\left[\begin{array}{cccc} 1 & & & \\ & 1 & & \\ & & \ddots & \\ & & & 1 \end{array}\right] \end{array}$$

(2)  *W* is a diagonal matrix of order *n*, where *ω*_*i*_  (1 ≤ *i* ≤ *n*) is the weight of *ith* category, and not all *ω*_*i*_ are equal to the others.

(14) $$\begin{array}{c} W=\left[\begin{array}{cccc} \omega_{1} & & & \\ & \omega_{2} & & \\ & & \ddots & \\ & & & \omega_{n} \end{array}\right] \end{array}$$

We consider two situations of weight matrix *W* in this paper.

Calculating weight is the key element that gives a weight to various features based on certain principles. As different features have different discriminative abilities on specific emotions [34], for weight definition, we adopt feedback strategy. Firstly, recognition results are obtained from the above-mentioned methods that used separate classifier for each physiological signal. Each emotion recognition rate of each classifier is ${\vec{P}}_{i}=(p_{i1},\ldots ,p_{im}{)}^{T}\;\;(1\leq i\leq n)$ treated as a weight matrix *W*_*i*_  (1 ≤ *i* ≤ *n*) as follows:

(15) $$\begin{array}{c} W_{i}=\left[\begin{array}{ccc} p_{i1} & \cdots & 0\\ \vdots & \ddots & \vdots \\ 0 & \cdots & p_{ij} \end{array}\right] \end{array}$$

In particular, from each classifier we obtained a weight matrix for each sentiment classifier. Secondly, let ${\vec{C}}_{i}=(c_{i1},\ldots ,c_{im}{)}^{T}\;\;(1\leq i\leq n)$ as the classifier probabilities resulted from each physiological signal are an m-dimensional vector, where $\left|{\vec{C}}_{i}\right|=1$ and *c*_*ij*_ ∈ {0,1}  (1 ≤ *i* ≤ *n*, 1 ≤ *j* ≤ *m*). And the classifier result $\vec{C}$ is obtained according to linear data fusion principle as follows:

(16) C=∑i=1nCiWi=∑i=1nci1⋮cimpi1⋯0⋮⋱⋮0⋯pij=∑i=1nci1pi1⋮∑i=1ncimpim

Finally, the recognition result is using a max-win strategy as follows:

(17) $$\begin{array}{c} \overset{m}{\underset{j=1}{max}}\left\{\;\overset{n}{\underset{i=1}{\sum }}c_{ij}p_{ij}\;\right\}=\overset{n}{\underset{i=1}{\sum }}c_{ik}p_{ik} \end{array}$$

And the most likely category label is *k*.

## 4. Experiment Result

**Figure 4:**
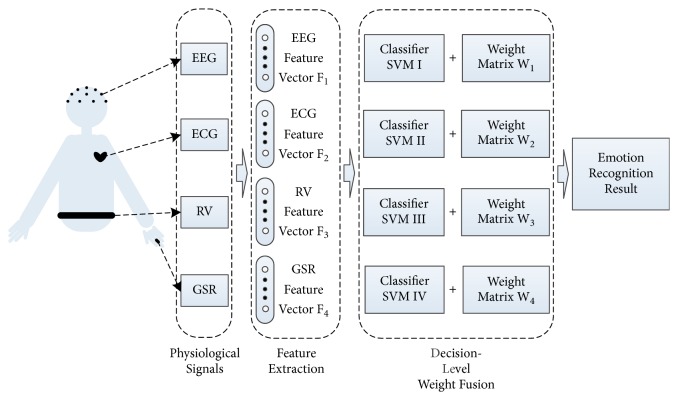
Flow of the emotional recognition based multichannel physiological signal.

The experiments on the MAHNOB-HCI database [35] show the effectiveness of the proposed method. In our experiments, we use EEGLAB, MATLAB, and python programs based on LIBSVM software packages, and the platform of data processing is a computer with Windows 7, Intel^(R)^ Core™ i3-2120 CPU (3.30GHz,) 4.00GB RAM. The flow chart of the experiment is shown in Figure 4, and specific steps are described in the following sections.

### 4.1. Experiment Data

MAHNOB-HCI is a multimodal database recorded in response to affective stimuli with the goal of emotion recognition. The recorded signals are shown in Table 1, we select the signals with italics in our experiments. The database records physiological signals of 30 participants with 9 different emotion labels. 30 volunteer participants have different cultural and education backgrounds.

Data recorded from 3 participants are not analyzed due to technical problems and unfinished data collection, so only 27 sets of data can be used in our research. The emotion labels are shown in Table 2, and we only use the data of labels with italics in our research. Concerning the imbalance of the emotion data set used in experiments, the size of training data sets is 80% of smallest emotion data. Besides, we use the remaining to test the classifiers, and the detailed number of data of each discrete emotion is shown in Table 3.

### 4.2. Results of Emotion Recognition

We extract each physiological signal emotion feature listed in Section 3 and apply the SVM classification independently. A comparison of results between each emotion has been shown in Table 4 for each physiological signal, respectively. Here, we observe that the emotional state of Neutral and Happiness are relatively easy to distinguish. And the highest average emotion recognition accuracy of 74.52% was obtained in EEG case. However, recognition accuracy of disgust is lower than ECG case. Besides, we can obtain emotion expression of each physiological signal which can be ranked according to the recognition rate and has been shown in Table 5. Obviously, various physiological signals have different abilities to classify specific emotion. Therefore, each result of physiological signal should be combined in a way that they benefit the interrelationships between the individual classifier.

We use the recognition rates in Table 4 and (15) to obtain the weight matrix of each classifier under the situation of diagonal matrix as follows:

(18) W1=0.66000000.76000000.50000000.86000000.63W2=0.55000000.67000000.69000000.78000000.50W3=0.45000000.55000000.46000000.63000000.38W4=0.47000000.53000000.50000000.69000000.50

Besides, we use (13) to obtain the weight matrix of each classifier under the situation of identity matrix as follows:

(19) $$\begin{array}{c} W_{i}^{\prime }=\left[\begin{array}{ccccc} 1 & 0 & 0 & 0 & 0\\ 0 & 1 & 0 & 0 & 0\\ 0 & 0 & 1 & 0 & 0\\ 0 & 0 & 0 & 1 & 0\\ 0 & 0 & 0 & 0 & 1 \end{array}\right],\;1\leq i\leq 4 \end{array}$$

Our proposed fusion frame is performed to combine the classification results of these four physiological signals. Thus we verify the fusion frame on the same training set and test set with two situations of weight matrix, respectively. A comparison of results between each emotion has been shown in Table 6 for each situation.

### 4.3. Results Analysis

We could see that the average recognition correct rate by using proposed method is 84.6%. And the recognition rate of each emotion is more than the accuracy of each individual physiological signal and under the situation of identity weight matrix. These confirm the effectiveness of our method. Investigating its reason, it can be explained from robustness of weighted fusion strategy. This method reduces the influence of weak correlation feature and enhances the influence of strong correlation feature by weighted feature, thus improving the robustness of classification algorithm. In brief, the method improves the accuracy of emotion recognition by giving full play to the advantages of various physiological signals and decision-level weighted fusion strategy and makes the whole fusion process close to human emotion recognition.

## 5. Conclusion

In this paper, we propose an approach of emotion recognition based on weighted fusion strategy of multichannel emotion data. In our work, single channel emotion recognition systems that use signal physiological signal were analyzed separately and tested with the same emotion databases. Then, physiological signals were used together with a weighted decision-level fusion. Weighted strategy is based on the effect of each physiological signal on various emotion recognition result is different. Thus we calculate weight of physiological signals based on their respective recognition rate and design a recognition model based on multichannel physiological signals. And recognition rate of each emotion is more than the accuracy of each individual physiological signal. Thus the experimental results suggest that the approach based on weighted fusion strategy has good performance on the correct rate in emotion recognition. In future work improvement of feature extraction strategy is probably the best avenue to enhance classification performance. Moreover, additional feature selection technique will be implemented to reduce the number of features and ameliorate the classification accuracies. Thus emotion recognition based on multichannel emotion data is still full of challenges in the future.

## Figures and Tables

**Table 1:** MAHNOB-HCI database recorded signals.

Emotion Data Modalities
*32-channel EEG (256 Hz)*
*3-channel ECG (256 Hz)*
*1-channel RA (256 Hz)*
*1-channel GSR (256 Hz)*
1-channel Skin Temperature (SKT) (256 Hz)
Face and Body Video (6 cameras, 60f/s)
Eye Gaze (60 Hz)
Audio (44.1 kHz)

**Table 2:** MAHNOB-HCI database recorded emotions and the corresponding labels.

**Label**	**Emotion**
***1***	*Sadness*
***2***	*Joy, Happiness*
***3***	*Disgust*
***4***	*Neutral*
**5**	Amusement
**6**	Anger
***7***	*Fear *
**8**	Surprise
**9**	Anxiety

**Table 3:** The size of each set of each emotion.

**Emotion**	**Sample Set**	**Training Set**	**Test Set**
**Sadness**	69	31	38
**Happiness**	86	31	55
**Disgust**	57	31	26
**Neutral**	112	31	81
**Fear **	39	31	8

**Table 4:** The detailed number of correctly recognized data and recognition rate under various physiological signals.

	**Sadness**	**Happiness**	**Disgust**	**Neutral**	**Fear**	**Average Recognition Rate**
**Test Set**	38	55	26	81	8	208

**EEG**	25	42	13	70	5	155
	**65.79**%	**76.36**%	50.00%	**86.42**%	**62.50**%	**74.52**%

**ECG**	21	37	18	63	4	143
	55.26%	67.27%	**69.23**%	77.78%	50.00%	68.75%

**RA**	17	30	12	51	3	113
	44.74%	54.55%	46.15%	62.96%	37.50%	54.33%

**GSR**	18	29	13	56	4	120
	47.37%	52.73%	50.00%	69.14%	50.00%	57.69%

**Table 5:** Emotion expression ordering of each physiological signal.

	**Emotion Expression Ordering**
**EEG**	Neutral > Happiness > Sadness > Fear > Disgust
**ECG**	Neutral > Disgust >Happiness > Sadness > Fear
**RV**	Neutral > Happiness > Disgust > Sadness > Fear
**GSR**	Neutral > Happiness > Disgust = Fear > Sadness

**Table 6:** The detailed number of correctly recognized data and recognition rate under two situations of weight matrix.

	**Sadness**	**Happiness**	**Disgust**	**Neutral**	**Fear**	**Average Recognition Rate**
**Test Set**	38	55	26	81	8	208

**Identity Matrix**	25	43	18	72	5	155
	65.79%	78.18%	69.23%	88.89%	62.50%	74.52%

**Diagonal Matrix**	28	47	20	75	6	176
	**73.68**%	**85.45**%	**76.92**%	**92.59**%	**75.00**%	**84.62**%

## References

[1] Cavallo F., Semeraro F., Fiorini L., Magyar G., Sinčák P., Dario P. (2018). Emotion Modelling for Social Robotics Applications: A Review. *Journal of Bionic Engineering*.

[2] Tojo T., Ono O., Noh N. B., Yusof R. (2018). Interactive Tutor Robot for Collaborative e-Learning System. *Electrical Engineering in Japan*.

[3] Basiri M., Schill F., U.Lima P., Floreano D. (2018). Localization of emergency acoustic sources by micro aerial vehicles. *Journal of Field Robotics*.

[4] Díez J. A., Blanco A., Catalán J. M., Badesa F. J., Lledó L. D., García-Aracil N. (2018). Hand exoskeleton for rehabilitation therapies with integrated optical force sensor. *Advances in Mechanical Engineering*.

[5] You L. Z., Zhang S. D., D Zhu L. Bed-chair integration-new developing trend of helpage assistive robot.

[6] Wioleta S. Using physiological signals for emotion recognition.

[7] Balters S., Steinert M. (2017). Capturing emotion reactivity through physiology measurement as a foundation for affective engineering in engineering design science and engineering practices. *Journal of Intelligent Manufacturing*.

[8] Ekman P., Friesen W. V., O'Sullivan M. (1987). Universals and cultural differences in the judgments of facial expressions of emotion. *Journal of Personality and Social Psychology*.

[9] Lang P. J. (1995). The emotion probe. Studies of motivation and attention. *American Psychologist*.

[10] Zheng W.-L., Lu B.-L. (2015). Investigating critical frequency bands and channels for eeg-based emotion recognition with deep neural networks. *IEEE Transactions on Autonomous Mental Development*.

[11] Guo H. W., Huang Y. S., Chien J. C., Shieh J. S. Short-term analysis of heart rate variability for emotion recognition via a wearable ECG device.

[12] Zhang Q., Lai X., Liu G. Emotion Recognition of GSR Based on an Improved Quantum Neural Network.

[13] Kim J., André E. (2008). Emotion recognition based on physiological changes in music listening. *IEEE Transactions on Pattern Analysis and Machine Intelligence*.

[14] Li C., Xu C., Feng Z. (2016). Analysis of physiological for emotion recognition with the IRS model. *Neurocomputing*.

[15] Wiem M. B., Lachiri Z. Emotion assessing using valence-arousal evaluation based on peripheral physiological signals and support vector machine.

[16] Chen J., Chen Z., Chi Z., Fu H. Emotion recognition in the wild with feature fusion and multiple kernel learning.

[17] Liu M. Combining multiple kernel methods on iemannian manifold for emotion recognition in the wild.

[18] Meudt S., Zharkov D., Kächele M., Schwenker F. Multi classifier systems and forward backward feature selection algorithms to classify emotional coloured speech.

[19] Liu M., Wang R., Huang Z., Shan S., Chen X. Partial least squares regression on grassmannian manifold for emotion recognition.

[20] Chen J., Hu B., Xu L., Moore P., Su Y. Feature-level fusion of multimodal physiological signals for emotion recognition.

[21] Khezri M., Firoozabadi M., Sharafat A. R. (2015). Reliable emotion recognition system based on dynamic adaptive fusion of forehead biopotentials and physiological signals. *Computer Methods and Programs in Biomedicine*.

[22] Lin Y.-P., Wang C.-H., Jung T.-P. (2010). EEG-based emotion recognition in music listening. *IEEE Transactions on Biomedical Engineering*.

[23] Chang C.-Y., Chang C.-W., Zheng J.-Y., Chung P.-C. (2013). Physiological emotion analysis using support vector regression. *Neurocomputing*.

[24] Park M.-S., Oh H.-S., Jeong H., Sohn J.-H. EEG-based emotion recogntion during emotionally evocative films.

[25] Kapoor A., Burleson W., Picard R. W. (2007). Automatic prediction of frustration. *International Journal of Human-Computer Studies*.

[26] Kotsiantis S. B., Zaharakis I. D., Pintelas P. E. (2006). Machine learning: A review of classification and combining techniques. *Artificial Intelligence Review*.

[27] Kotsiantis S. B. (2007). Supervised machine learning: a review of classification techniques. *Informatica*.

[28] Sarkheil P., Goebe R., Schneider F., Mathiak K. (2013). Emotion unfolded by motion: A role for parietal lobe in decoding dynamic facial expressions. *Social Cognitive and Affective Neuroscience*.

[29] Zhang J., Chen M., Zhao S., Hu S., Shi Z., Cao Y. (2016). ReliefF-Based EEG Sensor Selection Methods for Emotion Recognition. *Sensors*.

[30] Tatum W. O., Ellen R. (2014). Grass Lecture: Extraordinary EEG. *The Neurodiagnostic Journal*.

[31] Ktata S., Ouni K., Ellouze N. ECG signal maxima detection using wavelet transform.

[32] Das P., Khasnobish A., Tibarewala D. N. Emotion recognition employing ECG and GSR signals as markers of ANS.

[33] Ahujaet N. D. GSR and HRA: its application in clinical diagnosis.

[34] Sun B., Li L., Zuo T., Chen Y., Zhou G., Wu X. Combining Multimodal Features with Hierarchical Classifier Fusion for Emotion Recognition in the Wild.

[35] Soleymani M., Lichtenauer J., Pun T., Pantic M. (2012). A multimodal database for affect recognition and implicit tagging. *IEEE Transactions on Affective Computing*.

